# School psychology and institutional initiatives for school inclusion processes

**DOI:** 10.3389/fpsyg.2026.1698658

**Published:** 2026-03-13

**Authors:** Pollianna Galvão, Leonardo Vieira Nunes, Aline Beckmann Menezes, Najda Maria Vieira da Silva, Paula Braga Kenyon, Mônica dos Santos de Oliveira, Claisy Marinho-Araujo

**Affiliations:** 1 Postgraduate Program in Psychology, Federal University of Maranhão, São Luis, Brazil; 2 Laboratory of Assessment, Research and Intervention in Autism Spectrum Disorder - LAPITEA, Ceuma University, São Luis, Brazil; 3 Postgraduate Program in Developmental and School Psychology, University of Brasilia, Brasilia, Brazil; 4 Postgraduate Program in Behavioral Theory and Research, Federal University of Pará, Belem, Brazil; 5 Postgraduate Program in Psychology, Federal University of Alagoas, Maceió, Brazil; 6 Northeastern University Boston, Boston, MA, United States

**Keywords:** education, human development, inclusion, institutional practice, school psychology

## Abstract

The aim of this article is to discuss inclusion from the perspective of critical school psychology, highlighting institutional initiatives to understand and construct a more plural, interdisciplinary and inclusive school environment. Initially, conceptions and concepts on the subject were reviewed in their historical and social processes, relating them to educational policies and to the possibilities for the school psychologist to act in order to raise awareness and to develop subjects in anti-capacity practices. Subsequently examples of the school psychologist’s work are addressed as a feasibility of professional practice to foster inclusion in educational spaces. Finally, it is advocated that critical school psychology be committed to establishing institutional and collective actions to consolidate pedagogical work in inclusive contexts, in order to enhance learning processes, communication and coexistence between different people. These actions expand the possibilities for complex psychological development for everyone - people with and without disabilities.

## 1 Introduction

Contemporary School Psychology has been grounded on research and professional practices based on the paradigms of Critical Psychology (Pavón-Cuéllar, 2019), which challenge the traditional perspectives in Psychology by questioning the emphasis and understanding of psychological phenomena in an individualistic and reductionist manner. Social, political, and economic factors should emerge as revolutionary and fundamental explanations of the subjectivity and of the historical materiality of human relationships in our society. The criteria discussed for selecting theories, policies and experience reports in this manuscript are broadly discussed since 1994 by professors and researchers from the School Psychology working group (grupo de trabalho – GT) from the National Association for Research in Graduate Studies in Psychology (Associação Nacional de Pesquisa em Pós-graduação em Psicologia – ANPEPP) in Brazil. The purpose of the working group is developing and consolidating School Psychology as a scientific field, which produces knowledge, research and intervention, characterized by plurality, diversity and complexity. Over decades, the researches produce and disseminate studies, reflections, theories, practices at the interface between School Psychology and Education, from the fundamentals and scientific evidences, defending the promotion of citizenship and rights of all people.

In line with the call for a critical perspective, School Psychology has been advocating a more pluralistic, interdisciplinary and inclusive professional practice. For Marinho-Araujo (2014, 2015), the institutional and collective approach embodies critical intervention, as it steers the focus of the school psychologist’s work toward raising awareness within the school collective, contextualized and mediated by relationships and semiotic processes of subjectivation that dialectically resignify the different actors and their actions. Institutional action, seeking critical and political engagement of subjects in subjective and sociocultural transformations, is anchored in the epistemological and theoretical contributions of Historical-Cultural Psychology (Vygotsky, 2000), which defends human development originating from the symbolic mediation between social history and individual, concrete background, shared through experiences in specific social development situations - a process amalgamated by historical influences and sociocultural relations shared between subjects (Marinho-Araujo, 2015).

Based on this perspective, the school psychologist can act as a mediator of more complex psychological development, encompassing everyone from students to the teaching staff, families, and other socio-institutional players. Counteracting individualizing, adaptationist, normative, exclusionary, and sometimes prejudiced actions, they can build a sense of belonging to the educational context that will allow them to map relationships, spaces, times, actions, beliefs, concepts, and dynamics; develop a sensitivity to listening to the institutional discourses and the “voices of the school”; build collective and institutional partnerships and commitments; provoke the redefinition of demands and create new spaces for dialogue and the circulation of the subjects’ discourses (Marinho-Araujo, 2014).

The proposal for institutional intervention in School Psychology, supported by Marinho-Araujo (2014, 2015), advocates the process of raising awareness as an important pillar of critical School Psychology work, as it constitutes a powerful political action in overcoming the conservative view of adapting or psychologizing psychological practices in an educational framework. This proposal is characterized by dynamic, participatory, and collective action operating systematically in the daily life of the institution and is anchored in four dimensions: Institutional Mapping, Psychological Listening, Consulting for Collective Work, and Monitoring the Teaching-Learning Process (Marinho-Araujo, 2014, 2015). These dimensions are not presented as hierarchical steps on a sequential scale of priorities. They must occur interdependently and in an integrated manner, articulated with the reality and the routine of the school, dialectically re-signified according to their relationships and contexts, times, and spaces, considering personal and professional characteristics, throughout the psychological intervention. Inherent to all the Psychology activities in schools, those activities must also be planned together with the educational institution’s managers, teams and professionals.

Considering that Critical School Psychology, based on such assumptions and foundations, favors intentional action to break up with traditional normative, coercive, and exclusionary paradigms, the objective of this theoretical article was to present the specificities of institutional psychological action in the face of the inclusion of people with disabilities. Concepts, conceptions, contextualization, historical updates, educational policies, and innovative possibilities for action are addressed in the sections hereafter. In addition, authors such as Huljev et al. (2024), from an international perspective, argue that the difficulties faced by children with atypical development should be addressed and supported by several actors at school, including teachers, peers, principals, parents, specialized professionals who intervene with inclusion practices.

It was presented the Institutional Intervention from Marinho-Araujo (2014, 2015) with the purpose of political action in School Psychology already broadly disseminated in the practice of psychologists in Brazil. This paper represents a theoretical essay in which it was presented an intellectual proposal about inclusion practices to students with atypical development, in addition to presenting in the end intervention policies in the federal district, implemented by school psychologists in public schools from the region for over 5 decades.

## 2 School inclusion: concept, conceptions, updates

Historically, formal education has been available to privileged groups. Among the various segments of society excluded from the school benefit, people with disabilities stand out; this is a target group for special education in Brazil. Mendes (2006), when describing the schooling trajectory of this group, points out that the 16th century marks the beginning of these initiatives, but with an institutionalizing and segregating nature, under a welfare-based and custodial discourse. Over time, several advances have been made toward ensuring rights and access to education due to the struggle of social movements led by people with disabilities, their families, and professionals active in this field. Even so, there have been recurring historical episodes in which this insertion into the educational framework occurred without any support or modification of the school environment, imposing individual adaptations or inevitable dropouts.

Alves and Mol (2021) approach inclusion as an “educational, social and political movement that aims to defend the participation of all individuals, in an integral and responsible manner, in the society of which they are a part, with their differences being accepted and respected” (p. 5). It must be understood that accepting and respecting are attitudes that imply guaranteeing access to rights and, within the school environment, providing conditions for the individual development of potential, citizenship, and subjectivity. Thus, when speaking of an inclusive school, we are advocating practices aimed at welcoming ethnic-racial, religious, sexual, gender, socioeconomic, and other diversities. It can be stated, therefore, that the scope of inclusion used in this article, limited to people with disabilities, addresses only one strand among many that it would be possible to include.

Ferreira (2023) emphasizes that the understanding of disability varies throughout history and carries with it social and political implications. Currently, the definition of disability is predominantly based worldwide on the social model, which is associated with the physical aspect of perception by society. That is, what characterizes disability is not the individual’s organic constitution, but rather the extent to which society is able to facilitate disabled individuals’ development and the full exercise of their citizenship (Gesser et al., 2020). This perspective generates political implications by understanding disability as a sociological phenomenon, albeit inexorably linked to individual biological functioning—consistent with the defectology studies developed by Vygotsky (2000).

This way of defining and understanding disability sets a debate within the domain of human rights rather than biology. Matos (2020) argues that disability is, therefore, a product of social organization and individual-society relationships. Thus, the need for school inclusion emerges to curb the oppression and exclusion that have historically surrounded disability.

Alves and Mol (2021) corroborate this perspective, arguing that school inclusion is a student’s right, but that it can only occur if there are changes in schools covering the physical structure, management systems, classrooms, and the training of education professionals. A school prepared for diversity and respect for the individual pace of its students enables a reduction in the barriers faced and, consequently, in disabilities, as advocated by the social model of disability.

The number of students with disabilities enrolled in regular schools increased tenfold between 2002 (110,536 enrollments) and 2019 (1,090,805) (Brazil, 2019), largely as a result of the Brazil (2008). This increase in the number of students with disabilities triggered a series of demands on schools to adapt to the new reality – diverse and plural – from both a structural and political, social, and pedagogical perspective. This adaptation also implied the need to better refine the responsibilities of professionals who deal directly with school inclusion (Lopes and Mendes, 2023).

For Matos (2020), the transformation of school reality cannot be disconnected from a historical and political contextualization that promotes the problematization of the reality in which we live, with its inequalities and injustices. Thus, that author argues that professionals who will work in school inclusion must inevitably seek appropriate qualifications and ensure that the “focus of actions must be on society’s deficiencies, the empowerment of individuals in disadvantaged situations, and diversified human support” (Matos, 2020, p. 108). One professional who acquires a specific role in this struggle to foster school inclusion is the school psychologist. According to Matos (2020), Psychology can contribute to the understanding of the educational process, identifying mechanisms of exclusion and contributing to interventions that promote coexistence and learning in the context of diversity.

Galvão et al. (2024) report multi-methodological interventions to promote school inclusion, specifically related to children with autism spectrum disorder (ASD). Among several aspects described in the reports, it is worth highlighting Institutional Mapping, a dimension of Marinho-Araujo's (2014, 2015) institutional action proposal, involving contact with the classroom space, with the children themselves, with parents, teachers, and members of the pedagogical team. The authors also advocate the role of the School Psychologist in advising the school community as a whole, providing support and settling family concerns, mediating training processes, and implementing “scientifically based interventions to boost child development at the social and academic levels” (Galvão et al., 2024, p. 23).

In line with this discussion, Alves and Mol (2021), based on a literature review, state that for School Psychologists’ work to be effective for inclusion in institutions, it is essential that these professionals be part of the school’s daily routine, contributing sociocultural elements (such as respect for differences and the creation of bonds) and facilitating the learning process. Furthermore, they must contribute to the training of other professionals working in the school, always based on the specificity of psychological knowledge and from a critical, sociohistorically constructed, and empowering perspective, so that the team becomes aligned around the same principles that underpin inclusion, such as justice and equity (Matos, 2020). Some possibilities for School Psychologists’ work in connection with school inclusion will be developed in the sections hereafter.

## 3 School psychology and school inclusion: innovative possibilities for action

### 3.1 School psychology in inclusive education: practices in the process of inclusion of autistic students in regular schools

This section aims at presenting possibilities for School Psychology to include autistic students in regular schools. The theoretical and methodological foundation of this work is based on the institutional intervention proposal of Critical School Psychology (Marinho-Araujo, 2014, 2015), whose epistemological affiliation considers social, historical, and cultural foundations as the genesis of typically human psychological phenomena, namely the specificities of ontogenetic development, with or without disabilities. Multi-methodological interventions aimed at autistic individuals are considered to encompass a diversity of possibilities for action in psychological science based on scientific evidence (Galvão et al., 2024).

Autism spectrum disorder (ASD) is considered a neurodevelopmental specificity. According to the diagnostic criteria for ASD defined by the Diagnostic and Statistical Manual of Mental Disorders, 5th edition - DSM-5 (American Psychiatric Association, 2013), there are two main domains to be considered: persistent deficits in social communication and social interaction; and restricted and repetitive patterns of behaviors, interests, or activities. These characteristics present in the human form of existence of the autistic person bring consequences to social interactions in different contexts, including schools, given the recent history of school inclusion policies (Borges et al., 2024; Schmidt et al., 2024). Since the implementation of Federal Law No. 12,764/2012 (Brazil, 2012), a national policy that establishes the protection of the rights of autistic people, it is understood that this human characteristic belongs to the group of people with disabilities given the specificities of neuropsychological functioning.

As a starting point, two reflections were selected regarding the role of school psychologists in the process of including autistic students. The first concerns the sociopolitical function of educational spaces. Formal education institutions in Brazil are considered instruments for reducing social injustices (Gesser et al., 2020; Borges et al., 2024), playing fundamental roles, including teaching curricular knowledge to develop competencies aimed at the graduate profile outlined in the educational guidelines, such as the National Common Curricular Base (Brazil, 2023). The other role is to shape individuals through the circulation of values, ideas, beliefs, and moral and ethical precepts for all, students with and without disabilities, with the clear conviction that everyone benefits from school inclusion. In this sense, we agree with Marinho-Araujo (2014) in the statement that “[...] the school space constitutes a privileged place where, if on the one hand contradictions and antagonisms are made explicit, on the other it is possible for more just, democratic and supportive social interests to be constituted and articulated” (p. 157).

The second point of reflection concerns the necessary profile of a school psychologist for the inclusion of autistic students, given the recent history of implementing school inclusion policies in Brazil and the daily challenges that signal potential indicators for a redefined and expanded role aligned with the ethical and political commitment to a democratic school (Marinho-Araujo, 2015). It is important that this professional contributes with an updated psychological knowledge to the understanding and assessment of the learning processes of students with autism and, consequently, mediates the inclusive educational practices recommended by the (Brazil, 2008) (National Special Education Policy in the Perspective of Inclusive Education) (Brazil, 2008) and the Brazilian Inclusion Law (Brazil, 2015).

In this sense, a perspective that has stood out in psychological science for defining methodologies and technologies for behavioral intervention in the collective inclusion of students with ASD is the Applied Behavior Analysis (ABA) (Cooper et al., 2007). ABA interventions are also focused on teaching and refining academic repertoires, which is relevant for improving the development of students with ASD in a regular school context (Galvão et al., 2024), in addition to developing behavioral technologies that involve school stakeholders to enhance the school inclusion of autistic students. (Borges et al., 2024; Schmidt et al., 2024).

Given the recent approval of CNE/CP Opinion No. 50/2023, on November 12, 2024, which establishes specific guidelines for students with ASD, challenges are renewed for the work of this professional. To include the ASD population in the regular education process, aiming to meet the guidelines of the recent Policy, the school psychologist must be even more sensitive to the daily tensions related to pedagogical decisions for a population with multiple diversities of being and difficulties in school adaptation, but also with possibilities and potential for development when their specific learning needs are met. Knowledge arising from Evidence-Based Practices (EBP) is relevant to addressing the characteristics of this population in educational institutions (Schmidt et al., 2024).

Detrich et al. (2008), in their seminal work, address the critical need to integrate evidence-based practices into the educational settings to improve student outcomes and the effectiveness of the educational systems. The authors emphasize the importance of using scientifically validated interventions and provide a detailed roadmap for navigating the complexities of translating research into practical strategies in schools. By exploring these components, the authors aim to bridge the gap between empirical research and practical application, ensuring that educational systems are guided by proven methodologies.

An example of evidence-based practices is Positive Behavioral Interventions and Supports (PBIS). PBIS was established to disseminate evidence-based behavioral interventions for students with behavioral problems. However, the focus shifted to school-level behavioral support for all students, with greater emphasis on implementation practices and systems. Thus, PBIS has eventually be defined as a structure or framework designed to improve the adoption and implementation of a continuum of evidence-based interventions, aiming to achieve important academic and behavioral outcomes for all students (Horner et al., 2015). PBIS operates on a Multi-Tiered System of Supports (MTSS) model: Tier 1: universal support for all students; Tier 2: support for at-risk groups; and Tier 3: intensive individualized support.

Based on these arguments, the institutional and preventive role of the school psychologist has been given priority, valuing the potential of individuals, encouraging the development of diverse teaching strategies, and fostering social transformations for a democratic and inclusive education (Marinho-Araujo, 2014, 2015). The psychologist’s role should focus on the mediation, development, and use of behavioral technologies directly in schools (Marinho-Araujo, 2014). In this mediation, it is up to the psychologist (1) to be open to dialogue with principles and practices of Behavior Analysis Applied to ASD, with teams that meet the criteria for supervised practice [that is, with professionals who meet the criteria for specific training in the area of intervention for atypical development (ABPMC - Associação Brasileira de Psicologia e Medicina Comportamental, 2017)], that is, a master’s and/or doctorate stricto sensu in Behavior Analysis with certification from CABA-Br (Certification of ABA-Based Intervention Service Providers for ASD/Atypical Development); and (2) to monitor the work of school professionals on a daily basis with view at reducing support, in the search for coherence in the execution of the school’s Political Pedagogical Project (PPP) for all, with analysis of the particularities of each student with ASD and consistent with the BNCC guidelines (Brazil, 2016).

It is understood that individualized monitoring of autistic students must occur within and by the collective school setting, so that those students can develop academic and social skills anticipated by the school’s human development project and follow their school and social routine with greater autonomy (Marinho-Araujo et al., 2022). The use of ABA principles such as discrimination, generalization, and procedures such as differential reinforcement, hierarchy of cues, and gradual fading of assistance in the school context can enhance the inclusion processes (Galvão et al., 2024, 2018; Schmidt et al., 2024). Assessments of the quality of social interactions at school between students with and without autism, as well as the implementation of collective and systematic strategies for behaviors considered challenging in students with ASD, should be carried out to better understand the dynamics of relationships among school stakeholders, removing the *a priori* focus on the idea that difficulties in school adaptation are concentrated solely on the autistic individual (Borges et al., 2024).

With these scientific emphases, strategies for mediating social interactions involve teachers, assistants, peers, and other school players, with the support of therapeutic helpers in supervised practice (Galvão et al., 2024); this way, the child/youngster with autism has his/her development assured by the institution with view at beefing up the ASD learner’s potential. The role of the school psychologist is necessary to bring about changes in the concepts of development and learning that support educational practices (Marinho-Araujo, 2015), especially in relation to the school inclusion of students with autism. It is argued that the presence of a psychologist can favor the process of inclusion of this student population, influencing pedagogical measures that contribute to their effective permanence in school. In this sense, the importance of complying with Law No. 13,935/2019 is reiterated, to ensure the hiring of school psychologists for the purpose of working daily in regular educational institutions, in view of the historical commitment of psychology to the quality of educational actions and the increase of human plurality and diversity in and for the construction of a democratic and inclusive school.

### 3.2 Interpersonal relationships with people with disabilities in the regular classroom: school psychology with the fostering of ethical responsibility for educational inclusion

It is a fact, as mentioned in previous sections, that more than 1 million students who have some kind of disability are enrolled in regular classrooms (Brazil, 2019). However, despite this being undeniably a political and social achievement, access to regular classrooms for people with disabilities does not achieve complete educational inclusion.

When assessing this situation, school psychology professionals who practice critical thinking (Marinho-Araujo, 2015) denounce the historical reductionism that has been propagated as an impediment to human development in the educational settings. According to those scholars, schools have been fertile ground for the dissemination of prejudiced, racist conceptions and the valorization of productive cognitive profiles, akin to ableism driven by the assumptions of the consumer society (Gesser et al., 2020). In this framework, people with disabilities, in order to get closer to normality, must undergo a metamorphosis with the aid of rehabilitation, whether medical, psychological, or educational.

The precarious nature of educational settings has challenged the initiatives of school psychologists focused on inclusion. In keeping with their fundamental political principles to foster human development and social transformation, these professionals have focused on discussions about the social model of disability (França, 2013).

There is currently a frequent demand for School Psychology services. A common approach in these situations is to use spaces known as resource rooms, offered as support for students with disabilities to achieve learning levels similar to their non-disabled peers. However, it is not difficult to acknowledge the distortion of the sense of inclusion that permeates this approach, as it weakens the concept of coexistence with diversity, a constitutive condition of human societies.

As a critical goal, these professionals strive to propose actions that fill a certain gap in the documented frameworks that conceive and regulate inclusion regarding issues related to interpersonal relationships and communication processes. These documents lack sufficient analysis, estimates, or guidance on the specificity of expected asymmetries in the exercise of communication and otherness, when considering the coexistence of individuals with or without disabilities.

The meaning of inclusion remains inconsistent for many school professionals, who are sometimes the same ones who adopt segregationist practices in their interventions aimed at students with disabilities. Thus, a distancing from concrete situations can be perceived, favoring generic guidelines, without names, without real people in their relationships. For this reason, there is still no investment with an appropriate political focus on the level of interpersonal relationships; in this case, inclusion would take a significant step forward, as it would encompass its bidirectional meaning. With the crossing of differences, it will be possible to also include people with disabilities, by exercising the role of those who produce knowledge about disability. In the classroom, this knowledge takes on a revolutionary character over the prevailing traditionalism, regulated by formal practices in the teaching and learning process fostered in higher education institutions and, especially, in the basic education ones.

This analysis underlies the assumption that School Psychology services, from a critical perspective, are aligned with a broader concept of inclusion and advance the realization of the right of access to schools for people with disabilities, fostering their development and that of all the students within the school community. With this characteristic, inclusion is a political and social movement (Alves and Mol, 2021) that establishes diversity as a constituent element of the human condition and is enhanced in the educational settings.

This discussion highlights the importance that people who are not disabled to know about disability when they experience situations of otherness similar to those in the educational settings. We point out that the conditions for this knowledge and its broad implications for educational inclusion are demands on the school psychology services that are committed to the transformation and humanization of society. Approaches to these demands are still little known or even still need to be developed. Therefore, explanations regarding the conceptual parameter of surplus vision are presented here to support methodologies within School Psychology aimed at developing ethical responsibility, assuming that these are implications of the communicative processes that constitute human interactions, which assume specificities in the crossing of the differences targeted by educational inclusion.

Investing in the production of this information demonstrates that the political and social nature of the school psychologists’ professional practice also presupposes a concern for the clarity of the epistemological foundations that guide pedagogical practices in inclusion. This clarity is necessary to provide reasons that justify the urgent need for a profound review of the objectives of formal education, which must be fully aligned with the broad recognition of the political and social role of schools.

### 3.3 School psychology and school inclusion – intervention policies in the federal district

In the Federal District, the Secretaria de Estado de Educação (SEEDF, State Department of Education) has retained school psychologists in public schools for over five decades to work in interdisciplinary teams, providing school support service. These professionals are hired through a public selection process to work full-time in a school, ensuring their presence and their work within institutional and collective perspectives (Governo do Distrito Federal/GDF, 2019).

SEEDF’s guide, mainly the Curriculum in Motion (Governo do Distrito Federal/GDF, 2014) and the Enrollment Strategy (Governo do Distrito Federal/GDF, 2024), are based on the premise that all schools in the public network are inclusive, ensuring that educational policies and their developments reach the school community in all its diversity and contexts. In this connection, schools must organize themselves to ensure access, reception and permanence of each student, regardless of ethnicity/race, gender, belief, age, disability, social condition or any other specific situation, complying with the subjects’ differences, needs and potential. The Curriculum in Motion (Governo do Distrito Federal/GDF, 2014), based on the theoretical-methodological frameworks of Historical-Critical Pedagogy and Historical-Cultural Psychology, directs that the school should establish foundations, objectives, goals and initiatives that guide the organization of pedagogical work, taking into account the cultural and social plurality and diversity that characterize society and educational spaces.

In line with the guidelines of the DF Department of Education and the Pedagogical Orientation (Governo do Distrito Federal/GDF, 2010), the work of the school psychologist, from a critical perspective, has been marked by intervention in the different regular school institutional spaces in public school networks, promoting reflection and expanding understanding of inclusive processes.

Among the actions developed by the school psychologists, those focused on collective action and those that aim to develop spaces for dialogue with educational stakeholders stand out; they foster the circulation of knowledge, perceptions, and meanings among students, in conjunction with the school’s pedagogical proposals. Pedagogical meetings with the school staff, coordination and planning sessions, meetings with families, and training activities are a few examples of possibilities for psychologists’ interventions in developing activities related to school inclusion.

The school psychologist participates in meetings to develop the school’s Political-Pedagogical Project (PPP) and, based on the institutions mapping (Marinho-Araujo, 2014, 2015), he/she identifies the educational demands and needs of the school community. The psychologist’s perception contributes to the recognition of human diversity in the school setting; it also shapes actions, interventions, and projects that consolidate inclusion practices in the different spaces of the institution. In addition to the debate and collective development of the PPP, the psychologist contributes, in this document, to the specific planning of actions that ensure inclusive activities in the school setting.

One of the actions outlined in the SEEDF documents regarding the inclusion of students with disabilities concerns the implementation of pedagogical adaptations, which range from the organization, presentation, and work with content to the planning and evaluation of pedagogical dynamics in the school setting. The school team, of which the school psychologist is an integral part, reviews intervention and curricular possibilities according to each student’s potential, establishing programs that foster their learning and development. The psychologist, at this stage, is responsible for presenting the foundations of Developmental and Learning Psychology, based on Historical-Cultural Psychology (Vygotsky, 1989, 2000), with the aim of problematizing and guiding interventions regarding the curriculum and relational and intersubjective processes, as well as identifying and disseminating practices for academic success.

Another important practice carried out by the school psychologists throughout the year concerns the Case Studies, which consist of meetings with the teaching team to review and make decisions regarding the procedures to be adopted for the students’ academic development, where the best approaches to meet their specific needs are proposed. This process, which is outlined in the Enrollment Strategy (Governo do Distrito Federal/GDF, 2024), enables planning actions for the current or subsequent school years, establishing class organization, services at school and in the external network, and other measures that ensure the continuity of the educational inclusion process and the students’ comprehensive development. In the Case Studies, the school psychologist contributes to the discussion on the learning assessment, from a procedural and dynamic perspective, in addition to prompting reflections and problematizations on topics relevant to students’ school life, such as the medicalization of lifestyles, family-school relationships, teaching and learning processes, and the dynamics and organization of the pedagogical work.

In the Class Councils, which are regular activities provided for in the SEEDF program, the school psychologist has been involved in the discussion on the development and learning of the subjects, redefining school complaints and evaluating inclusion processes for all. The analysis on the evaluation of learning is not restricted to the subjects individually, but involves the entire school setting and the pedagogical proposal provided for in the Political-Pedagogical Project, in direct articulation with the teaching practice and with the intervention projects that materialize in the school routine.

As a specific psychologist’s practice, psychological assessment must consider all factors involved in daily school life, as well as the institutional and pedagogical relationships related to the teaching and learning process. It is the professional’s responsibility to diversify the instruments and methods of observing the school context, both individually and collectively, considering what to evaluate, the type of information to be obtained, and the decisions to be made, providing support and guidance to the teachers and administrators in proposing pedagogical interventions. The psychologist, working with the school team, could encourage reflection on stigmas, prejudices, and difficulties in the inclusion process, systematizing actions to promote and develop these youngsters, with a view to education success.

There is also work with families, with various possibilities for interventions on the topic of inclusion that the school psychologist can carry out; this work may range from parent/guardian meetings to the creation of new spaces for this debate. Meetings, workshops, lectures, and support for families are organized so that they can raise questions, propose, and participate in the decisions that improve educational practices, as representatives of the school community. The psychologist plays the role of mediator and educator in the family-school relationship, practicing psychological listening (Marinho-Araujo, 2014, 2015) to understand the social and educational phenomena that are part of the school routine and intertwine with the topic of inclusion, highlighting the roles of the players involved in these relational dynamics.

The ongoing training of school professionals is a responsibility that permeates the other activities of the school psychologist, as all school settings provide opportunities for study, reflection, and analysis of the context in which pedagogical practices are performed. Within the specific scope of psychological knowledge, the psychologist provides systematically and regularly, training in the collective coordination meetings with the group of teachers, with school management, during meetings and the planning sessions, directing inclusion initiatives and with the entire technical and pedagogical team, in meetings and workshops that address topics related to the inclusion process, human rights, and diversity, interdependent with the processes of learning and school coexistence.

In addition to the school’s professional staff, it is imperative to recognize students as active subjects in their development and schooling, and it is the school psychologist’s responsibility to create spaces for student participation and empowerment. Assemblies, discussion groups, and projects are held to promote student engagement, the development of autonomy, critical thinking, responsibility, and a sense of collaboration with pedagogical proposals. These initiatives involve all students in the school, not just those with disabilities or disorders, aiming to raise awareness about recognizing differences and combating all forms of prejudice and discrimination.

## 4 Discussion

The feasibility of School Psychology’s work in connection with school inclusion, based on critical, social, and cultural assumptions, was presented in this article through a report of experience and interventions in a public education system, supported by policies, ongoing training, and a strong commitment to the institutional collective. Disability, understood here as closely related to the current social system, can become a condition for the development of diverse, unexplored possibilities, especially within the educational context (Vygotsky, 1989).

The intentional work of school psychologists should encourage institutional players to take over the breaking up with traditional paradigms in Psychology, especially in critically mediating concepts and potentialities in human development. Critical School Psychology should increasingly commit to building partnerships and collective initiatives so that the pedagogical organization in inclusive contexts enhances complex processes of learning, communication, and exchanges between different groups, expanding the possibilities for complex psychological development for all—people with and without disabilities.

When addressing the topic of inclusion, these intervention proposals allow individuals to review their reality, recognize the issues that impact relational dynamics, and see themselves as participants in solutions and innovations that improve daily school life. Psychologists should promote, intentionally and preventively, inclusive actions as an integral part of their pedagogical work, beyond *ad hoc* proposals. Their practice should contribute to raising awareness among individuals and groups, understanding the importance of public policies as a mechanism for access, equity, and social justice, ensuring that the educational players exercise their capabilities and transform their relationships and social practices.

The reflections and propositions raised in the current article may suggest future studies which encompass the development of scientific knowledge in Psychology and related fields based on Evidence Based Practices (EBP) to intervene with students diagnosed with ASD and other special education students (Schmidt et al., 2024).
